# Exploring faculty and student perceptions of online surgical teaching during the COVID-19 pandemic: A qualitative study

**DOI:** 10.4102/jcmsa.v4i1.408

**Published:** 2026-08-25

**Authors:** Sumayyah Ebrahim, Ruvashni Naidoo, Jessica L. Phillip, Jacqueline M. van Wyk

**Affiliations:** 1 Discipline of General Surgery, Nelson R Mandela School of Medicine, College of Health Sciences, University of KwaZulu-Natal, Durban, South Africa; 2 Health Systems Research Unit, South African Medical Research Council, Cape Town, South Africa; 3 Maternal, Adolescent and Child Health Institute (MatCH), Durban, South Africa; 4 Department of Clinical and Professional Practice, Nelson R Mandela School of Medicine, College of Health Sciences, University of KwaZulu-Natal, Durban, South Africa; 5 Department of Health Sciences Education, Faculty of Health Sciences, University of Cape Town, Cape Town, South Africa

**Keywords:** e-learning, online teaching and learning, blended learning, surgical education, medical education, engagement, medical students, undergraduate

## Abstract

**Background:**

The coronavirus disease 2019 (COVID-19) pandemic necessitated a rapid transition from contact-based to online surgical teaching for final-year undergraduate medical students. While online teaching offers benefits such as flexibility and accessibility, it raises concerns about the loss of clinical exposure, which is integral to surgical training. This study explored faculty (teaching staff) and student perceptions of an online surgical teaching and learning module implemented during the pandemic, examining perceived effectiveness and identifying challenges and areas for improvement.

**Methods:**

A qualitative descriptive study was conducted using purposive sampling. Nine virtual synchronous focus group discussions were conducted via Zoom, one with teaching staff and eight with students. Focus group discussions were audio-recorded and transcribed verbatim, and the data were later analysed using thematic content analysis.

**Results:**

Five themes were derived from the data: (1) perceptions of online teaching and learning effectiveness; (2) a lack of clinical exposure and preparedness for internship; (3) module structure and self-organisation; (4) information technology-related challenges; and (5) the way forward post-COVID-19. Both groups valued the module’s organisation, resource accessibility, content standardisation and alignment of outcomes, teaching and assessment. Negative aspects included limited clinical and procedural exposure and challenges with student engagement in live Zoom sessions.

**Conclusion:**

Online learning proved a feasible alternative during the COVID-19 pandemic, with numerous benefits; however, both faculty and students agreed that blended modes of instruction should be explored going forward.

**Contribution:**

This study contributes to the development of practices for integrating online teaching methods into General Surgery education.

## Introduction

The traditional curriculum for final-year undergraduate medical students in the Discipline of General Surgery at the University of KwaZulu-Natal (UKZN) includes a 6-week rotation at a regional or tertiary hospital. It includes bedside teaching, tutorials, small-group discussions, service learning during intakes and ward rounds. The emergency measures for remote learning that followed coronavirus disease 2019 (COVID-19) restrictions affected traditional, contact-based training methods for medical students and prompted the use of innovative teaching methods to continue the teaching programme and produce skilled graduates for the South African health system. From March 2020 to December 2021, all teaching and learning for final-year undergraduate surgery students at UKZN were transitioned to a virtual teaching platform.^1^ The virtual platform incorporated both synchronous and asynchronous activities to reinforce theoretical concepts, facilitate case-based learning and teach skills related to core surgical topics.^1^

**FIGURE 1:**
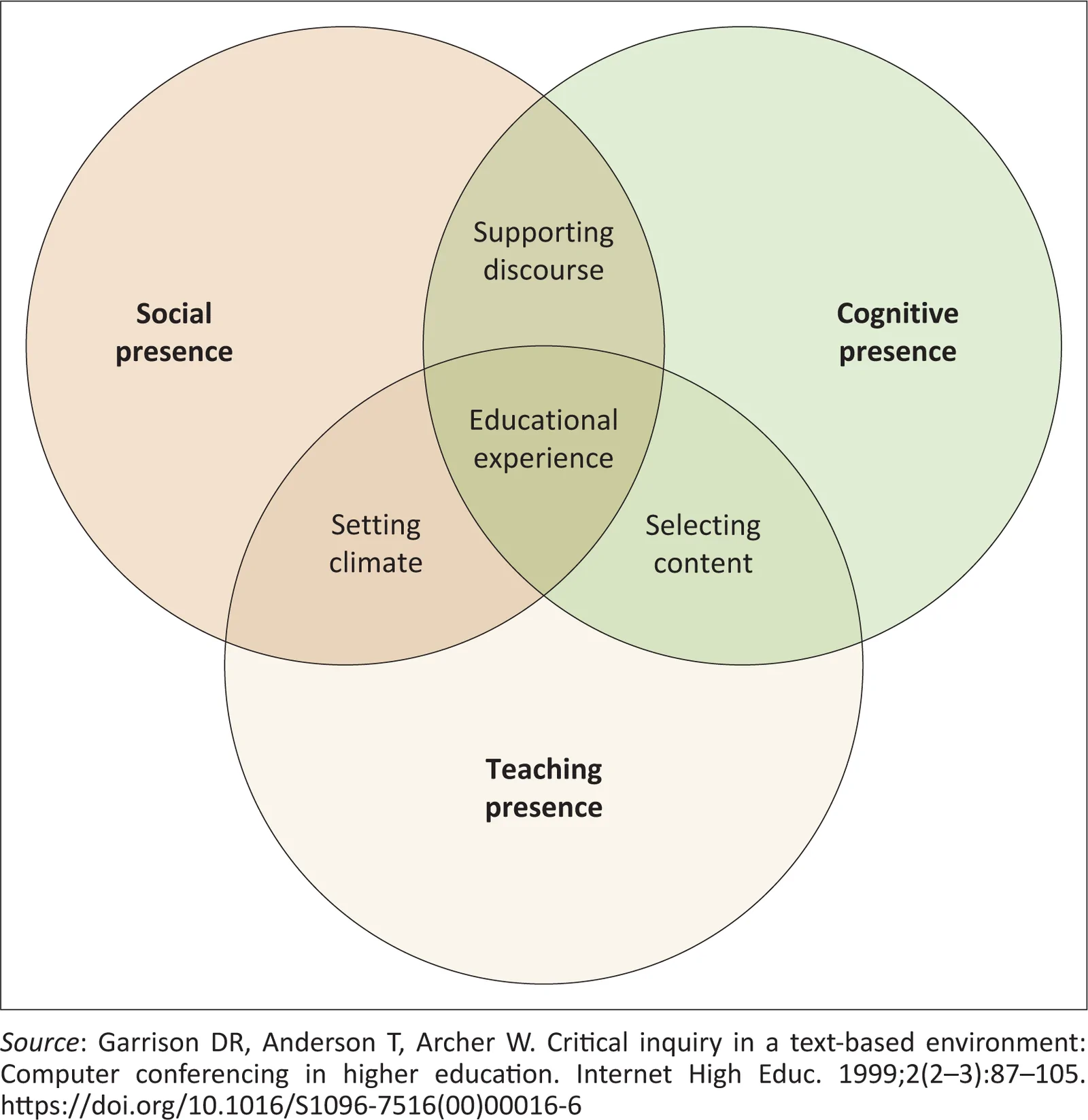
Community of inquiry framework for e-learning.

In keeping with adult learning theories as posited by Knowles et al., andragogy has five main principles: adult learners are self-directed, they have accumulated experiences that can be a rich resource for learning, become ready to learn when they experience a desire to know something, are problem-centred and are driven to learn by internal motivators.^2,3^ E-learning involves the ‘use of Internet and multimedia technology to deliver instruction and facilitate learning’.^4^ It is based on the overlapping educational theories arising from three main theoretical frameworks: behaviourism, cognitivism and constructivism.^4^ Garrison et al.^5^ developed the Community of Inquiry (CoI) framework for e-learning, based on three core, interrelated elements: cognitive, social and teaching presence (Figure 1). Cognitive presence is the ‘extent to which the participants can construct meaning through sustained communication’.^5^ Social presence refers to ‘the ability of learners to project themselves socially and emotionally, thereby being perceived as “real people” in mediated communication’.^5^ The last element, teaching presence, is ‘the design, facilitation and direction of cognitive and social processes to realise personally meaningful and educationally worthwhile learning outcomes’.^6^ Thus, the online CoI framework can be considered a collaborative constructivist model of teaching and learning.^7,8^

Factors that influence the effectiveness of online learning include technology characteristics such as access, navigation and an internet connection; the design of e-learning platforms and availability of both synchronous and asynchronous resources; instructors’ characteristics: teaching styles, attitudes towards students, digital skills and encouraging interaction in online sessions; and students’ characteristics, for example, digital knowledge and skills,^9,10^ and motivation and self-discipline.^11^

Perceptions are defined as ‘complex mental processes by which people understand, interpret, evaluate and form a picture of social phenomena’.^10^ Students’ perceptions of online learning have been found to affect their attention, motivation, emotions and satisfaction with learning.^10^ Thus, by studying student experiences of online learning, we can explore their perceptions and attitudes, which (when correlated with those of their instructors) can improve the quality of learning.^10^ Regarding perceptions of medical students towards online learning, evaluation of student feedback showed a preference for blended or hybrid teaching methods (i.e. a combination of face-to-face and online teaching), and positive feedback with high levels of satisfaction.^11,12,13^ Qualitative studies conducted during the COVID-19 pandemic in Saudi Arabia, the United Arab Emirates (UAE) and India that explored students’ perceptions of online learning identified advantages such as convenience, flexibility, time efficiency and the availability of recorded sessions.^14,15,16^ The described disadvantages included technical challenges (such as internet connectivity issues), the instructor’s inexperience, a lack of interaction and self-motivation and isolation and missed opportunities for practical learning.^14,15,16^

This study was conceptualised at the height of the COVID-19 pandemic. Based in a middle-income African country, we had little experience with online teaching in undergraduate surgery. Following the first year (2020) of online teaching and learning at our university, a qualitative study was conducted to explore faculty, that is, surgical teaching staff (consultants and medical officers) and student experiences in the online teaching and learning module, examining perceptions of effectiveness and identifying challenges and areas for improvement.

## Research methods and design

The features of the online surgical module developed for final-year General Surgery students at UKZN have been described before.^1^ In brief, the 6-week surgical module drew on flipped-classroom pedagogy, combining live synchronous sessions with asynchronous learning components,^1^ incorporating the three CoI framework elements, as shown in Table 1.

**TABLE 1:** Community of Inquiry categories, indicators and design of the online surgical module.

Elements	Categories	Indicators	Design of the online surgical module
Cognitive presence	Triggering event	Sense of puzzlement	Case-based, clinical scenario teaching and discussion, flipped classroom approach
	Exploration	Information exchange	Small group allocation to topics
	Integration	Connecting ideas	Synchronous ‘live’ Zoom tutorial discussion with tutors and peers
	Resolution	Apply new ideas	Weekly quizzes on Modular Object-Oriented Dynamic Learning Environment (Moodle) on course content, an end-of-block theory-based multiple-choice question (MCQ) paper and a clinical vignette paper-case assessment
Social presence	Emotional expression	Emotions	Limited because of ‘camera off’ during live Zoom sessions
	Open communication	Risk-free expression	Approachable tutors, students were able to ask questions and clarify concepts in live Zoom sessions
	Group cohesion	Encouraging collaboration	Small group allocation to topics, informal peer learning groups
Teaching presence	Design & organisation	Setting curriculum & activities	Structured, organised module. Synchronous Zoom tutorials and asynchronous activities on Moodle learning management system (LMS) (videos on clinical examination and procedural skills, interactive video quizzes, PowerPoint presentations and text resources)
	Facilitating discourse	Shaping constructive exchange	Live Zoom tutorial discussion with tutors and peers
	Direct instruction	Focusing & resolving issues	Revision sessions, being able to email tutors, and students were able to ask questions and clarify concepts in live Zoom sessions

*Source*: Garrison DR, Anderson T, Archer W. Critical inquiry in a text-based environment: Computer conferencing in higher education. Internet High Educ. 1999;2(2–3):87–105. https://doi.org/10.1016/S1096-7516(00)00016-6

### Study design

We used a qualitative descriptive research approach, grounded in an interpretivist paradigm. Interpretivism allows ‘individual perspectives to be heard and for education researchers to examine understandings from the perspective of those involved in a particular teaching and learning activity or environment’.^17^ In our setting, this enabled exploration of staff and student experiences and perceptions of the online teaching and learning module implemented at our university. We conducted virtual focus group discussions (FGDs) via Zoom, using semi-structured interview guides. Focus group discussions were chosen to facilitate an interactive, in-depth exploration of participants’ experiences. Group processes can encourage participants to explore and clarify their opinions in ways that would be less easily achievable in a one-on-one interview.^18^ Additionally, focus groups generate critical comments because of reinforcement and rebuttal by other participants, especially when peers and colleagues are present in naturally occurring groups.^15^

### Study setting and participants

In 2021, approximately 325 final-year medical students undertook a 6-week rotation in a combined General Surgery and Orthopaedics module. Twenty-five tutors provided live Zoom teaching sessions, comprising surgical consultants and medical officers who were staff members on the undergraduate surgical training module. A separate department administers the Orthopaedics component of the block. Thus, only comments related to the General Surgery component are considered in this study.

### Recruitment and sampling

A purposive sampling strategy was used, and email invitations were sent to all faculty who teach medical students to participate in the online FGD. At the end of each rotation, in a live Zoom session, final-year undergraduate medical students who had received only online teaching in Surgery were informed about the study’s purpose and invited to participate. Convenience and snowball sampling methods were used to recruit student participants. Emails were sent to students, and the student representative for each rotation was approached to help recruit more students via social networks. This approach was chosen because these students had direct contact with the wider student group. Focus group discussions were scheduled at a time convenient for students and facilitators. We aimed to create homogeneous groups to better express and understand shared experiences.^18^ Thus, separate FGDs were conducted with faculty, and student groups were kept separate according to their completed rotations.

### Research instrument and data collection

Two authors (Sumayyah Ebrahim and Jacqueline M. van Wyk), together with an experienced qualitative researcher (Jessica L. Phillip), developed a semi-structured FGD guide (Online Appendix 1 – Tutor and student FGD guides), guided by the Interview Protocol Refinement (IPR) framework.^19^ This framework informed a four-phase process: ensuring the FGD questions aligned with the study objectives, constructing an inquiry-based conversation, obtaining feedback on the draft guide and piloting the guide.^19^ The faculty and student FGD guides comprised four sections that probed their access to and experiences with the online teaching and learning module implemented, their perceptions of educational impact and effectiveness, the challenges they encountered and their recommendations. In keeping with the final phase of the IPR framework, the FGD guide for students was piloted with five students in April 2021. This exercise enabled us to refine our questions, reflect on the language used, gauge session duration and address confidentiality and anonymity concerns in virtual sessions.^20^ Two facilitators were present at each FGD; Sumayyah Ebrahim managed logistics, while Margy Matthews or Jacqueline M. van Wyk led the discussion. Data saturation was reached at the last FGD, and it was agreed that including more participants would not be necessary.^21^ The data collection methods are summarised in Table 2.

**TABLE 2:** Overview of the data collection methods.

Data collection strategy	Delivery	Description
Focus group discussions (FGD)	Nine synchronous virtual FGDs took place in 2021, comprising one faculty FGD and eight student FGDs. Prior to each FGD, participants received study information sheets and consent forms. FGDs lasted between 65 and 120 min and involved 67 participants in total: eight faculty members (two female, six male) and 59 students (32 female, 27 male).	FGDs enabled an in-depth exploration of faculty and student experiences within a peer group setting. Each FGD opened with introductions and a discussion of confidentiality; participants were reminded that participation was voluntary and that they could withdraw at any time without penalty. The sections of the FGD guide were outlined, and verbal consent was obtained for audio recording. Sessions concluded with a summary of key topics, an opportunity for closing remarks, and guidance on where students could access further educational resources if required. All FGDs were audio-recorded via Zoom, with transcripts subsequently edited and checked for accuracy. Field notes were additionally recorded.

### Focus group discussion team and reflexivity

Sumayyah Ebrahim, the lead researcher and module coordinator, participated in all FGDs. Her role encompassed inviting participants, distributing study information, collecting signed consent forms and providing technical support during sessions. As she was known to both faculty and student participants, two facilitators were required for each FGD. Jacqueline M. van Wyk, an associate professor from a different department at the university, was known to only one of the tutors and to none of the students; she co-facilitated the FGD with tutors. The second facilitator, Margy Matthews, was a medical doctor and a retired senior lecturer at the university. She led several of the student FGDs, as did Jacqueline M. van Wyk on occasion; both had prior experience in qualitative interviewing and were unfamiliar to the students. All three facilitators were female, whereas participants included both males and females. Faculty participants were well acquainted with one another, as were the student participants, through their shared clinical rotations.

### Data analysis

NVIVO 12 Pro software (QSR International, Melbourne, 2018) was used to organise, sort and code the data for the qualitative analysis. We conducted a thematic content analysis following the guidelines outlined by Braun and Clarke.^22^ A deductive approach guided the development of the coding framework, based on the study’s objectives and the FGD guide content. An inductive approach was also used to identify themes that were derived from the data.^23^ Subthemes were formed by consolidating similar codes, which were then organised under broader overarching themes. Two authors (Sumayyah Ebrahim and Jessica L. Phillip) independently followed the same initial coding and analysis steps, and their findings were then compared and discussed with co-authors (Jacqueline M. van Wyk and Ruvashni Naidoo) to reach consensus. This process of researcher triangulation reveals different perspectives, minimises individual biases and strengthens the credibility and overall trustworthiness of the study’s findings as described below.^24^

### Rigour

To ensure trustworthiness, the study addressed all four criteria of qualitative rigour.^24,25^ Credibility was supported through member checking during the FGDs and triangulation across faculty, student and prior quantitative data.^1,24,25^ Dependability was achieved via a documented audit trail of the recruitment, data collection and analysis process, along with external review by an independent observer (Jessica L. Phillip).^24,25^ Confirmability was strengthened through team-based coding input, direct participant quotations to substantiate findings, and researcher reflexivity, as described in the Methods and Limitations sections.^24,25^ Finally, transferability was noted as plausible to other higher-education settings in South Africa and comparable low- and middle-income countries.^24,25^ These criteria and their application are summarised in Online Appendix 1 Table 1-OA1.

### Ethical considerations

This study was conducted in accordance with the Declaration of Helsinki. Ethical clearance to conduct this study was obtained from the University of KwaZulu-Natal Biomedical Research Ethics Committee (No. BREC/00002686/2021). We obtained written informed consent from each participant. All FGD-related data, including the master participant list and consent forms, are held electronically on password-protected computers. Participants had the option of adopting a pseudonym (a false name) if they preferred. Participants were informed, both in the study information sheet and verbally at the start of each FGD, that participation was voluntary and that they could withdraw at any time without penalty. Participants’ names were anonymised in both the transcripts and the subsequent qualitative analysis. As participants were acquainted with one another, they may have discussed the session’s content afterwards; however, none of the topics covered was sensitive in nature. Comprehensive reporting was ensured through adherence to the Consolidated Criteria for Reporting Qualitative Research (COREQ) guidelines,^26^ as outlined in Online Appendix 1 Table 2-OA1.

## Results

**FIGURE 2:**
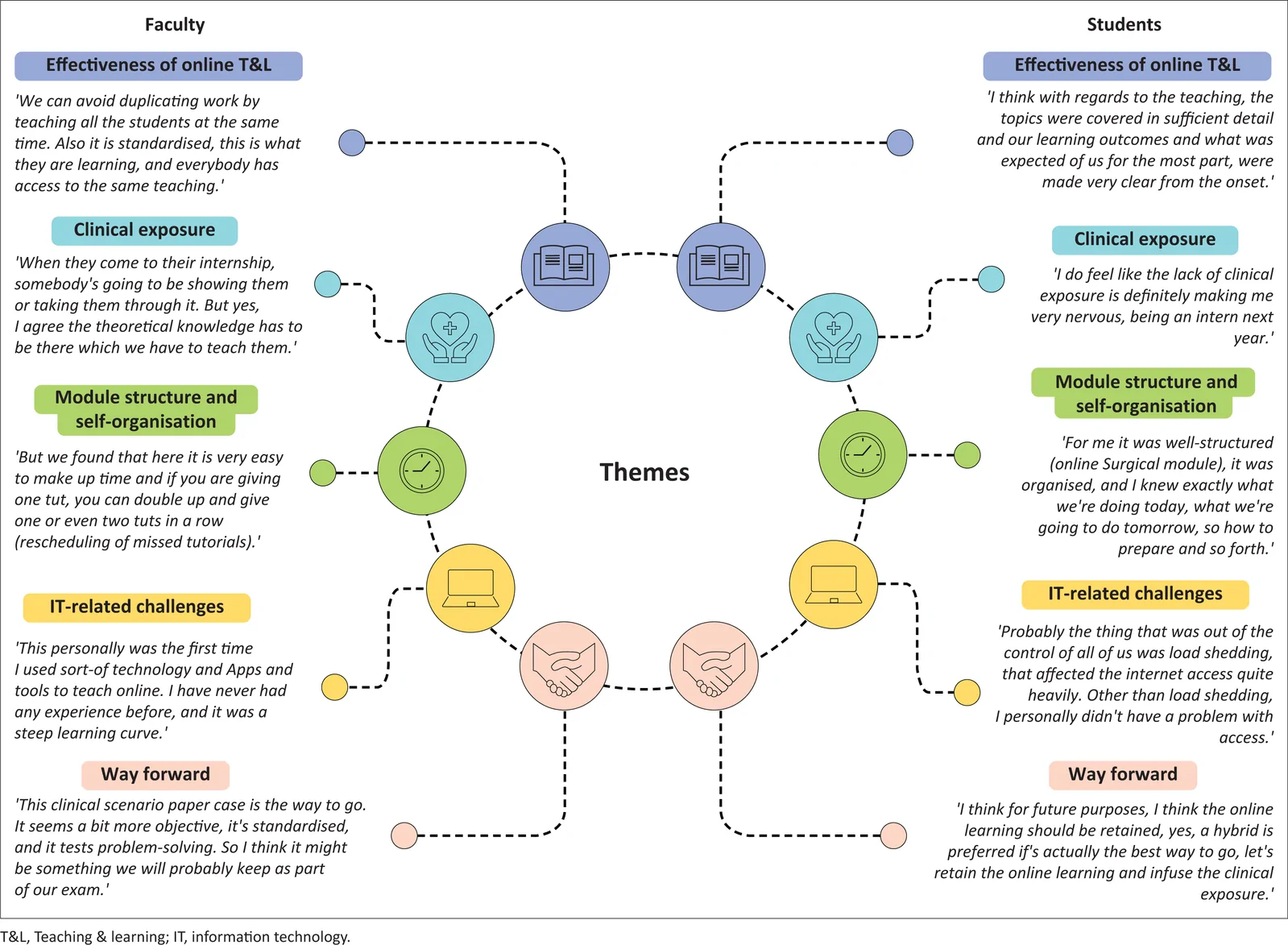
Major themes and feedback from focus group discussions.

Five major themes were derived from the data. These were: (1) perceptions of online teaching and learning effectiveness; (2) concerns about a lack of clinical exposure and subsequent preparedness for internship; (3) module structure and self-organisation; (4) information technology (IT)-related challenges and (5) the way forward post-COVID-19. In January 2021, we held the first FGD with faculty, and any recommendations to improve the online teaching programme were subsequently implemented. Student FGDs were held thereafter. The major themes, subthemes and representative quotes are summarised in Figure 2, and in Online Appendix 1 Table 3A-OA1 and Table 3B-OA1.

### Perceptions of the effectiveness of online teaching and learning

Most of the faculty involved in the online teaching programme had previously facilitated small-group bedside teaching and large-group in-person lectures. A major challenge identified by faculty during the transition to online teaching in 2020 was students’ poor engagement in the online environment. This made it difficult to identify students who may be struggling with concepts; however, for shy students, the Zoom platform may offer opportunities to ask questions via chat:

‘So basically, pre-COVID I was doing small group tutorials in one of the lecture theatres at Medical School on a weekly basis. And you know the advantage of that obviously, I think everyone is aware – you have immediate interaction, you can make eye contact with the students, and you can see whether they are having difficulty, understanding what you are saying and such the like. The problem with the Zoom platform is that I very rarely have students actually putting on their cameras. It is usually an uphill battle to even get them to put on their microphones. You ask a question, and there is deathly silence, so that makes a big difference, and that’s really the biggest advantage of face-to-face tutorials.’ (P2, male, tutor)

To promote greater student engagement and participation during synchronous Zoom sessions, faculty proposed assigning four or five students per topic to foster more active discussion.^1^ During these sessions, the students received immediate feedback, and their contributions were assessed against a rubric (Online Appendix 1 – Class Participation Mark Rubric), with the mark eventually contributing to their continuous assessment mark for the surgical rotation.

Students reported mixed feelings about the measures implemented to foster participation and engagement in the Zoom sessions. While they acknowledged that the system had the desired effect in motivating them to prepare on the topics allocated to them, and it obviated uncomfortable silences in the online sessions, for topics to which they were not allocated, they did not feel as well prepared, and this added to their anxiety when preparing for the end-of-block assessments. Students suggested that all students be allowed to participate in class discussions or that tutors use a random process to call on students, which will increase their motivation to prepare for all sessions:

‘There was a group allocated to present every day, so you leave everything to them. Like if they if they are covering melanoma today, there’s a specific group that has to answer questions for that, which means most of the work you don’t really have to study for it at the time which actually catch catches up later when you have to cover it for the exams so I was more focused on preparing for topics where I have to answer to, sort of neglecting the others, because I know I won’t be I won’t be asked any questions.’ (Student B, female, Year 6)

Other noted benefits include standardising teaching and avoiding duplication of work (Figure 2). The content was easily accessible on the Modular Object-Oriented Dynamic Learning Environment (Moodle) learning management system (LMS), which allowed for easy access to the content (lecture slides, previous videos of tutorials) before the session. This facilitated a flipped classroom approach, allowing tutors to use case-based scenarios to develop students’ clinical reasoning skills. Students reported that the case-based teaching approach to surgical pathology helped develop their clinical reasoning skills. Students also attributed their deeper understanding of course content to the dedication and patience of tutors, who took the time to repeat and clarify concepts until students felt confident that they had grasped them:

‘Most of the lectures we had were more like an approach to different topics. So, the way they delivered their lectures wasn’t like giving us information, but we had to give out as well. They would ask us the approach; what would you ask on history, what in the exam are you expecting and also the results interpretation of the results, the treatment. They tried to cover for the clinical part and also had enough clinical reasoning training.’ (Student B, female, Year 6)

‘The dedication of the lecturers. How they gave everyone enough time. The lecturers were incredible. They repeated things until you understood them and until you were able to [*explain things*]. I love the confidence that comes with the understanding that they actually gave us.’ (Student J, male, Year 6)

The assessment format for the clinical component also changed from a clinical case and oral viva voce examination before COVID-19 to a written, structured clinical case scenario examination. This format was found to be more objective and easier to administer via Moodle. As a high-stakes assessment, it was conducted in person in accordance with COVID-19 safety protocols. It also enabled faculty to mark remotely; however, because only a few tutors served as assessors, most tutors were unaware of how their students were performing on assessments and requested feedback to improve and/or amend their teaching. A tutor also suggested having a virtual examiner’s meeting following each rotation to discuss student marks and performance; before COVID-19, these meetings were held in person after each surgical rotation:

‘Also, when the students do go for exams at the end of their rotation, so we don’t know as well as tutors how well the students did in the exam? Are they understanding what we are teaching them? Are they doing well in the exams? I think also a little bit of feedback in terms of the exams. Previously, we were heavily involved in the exams we were there in the meetings so we knew if students were not doing well, and then we can improve on our tutoring strategy. I think that is another aspect to look at. It would be nice as well to know how well the students did in the exams. If they are doing poor, what do we need to improve on? I think maybe that is another aspect to look at.’ (P7, male, tutor)

### Lack of clinical exposure and subsequent preparedness for internship

One of the junior faculty members, a medical officer with post-specialisation training, expressed concern about a possible gap in the clinical and procedural skills training of final-year students:

‘Then (pre-COVID-19) we had smaller groups of 2 or 3 students allocated to our unit with whom we did bedside clinical teaching with, and we had them in our SOPD (Surgical Outpatient’s Department) as well, showing them how to do biopsies, examination, maybe approach to a breast patient or rectal exam, you know to walk them through that. I think that this is something that is now lacking with the Zoom tutorials.’ (P3, female, tutor)

However, another, more senior consultant argued that it was more important for students to have the requisite theoretical knowledge of how to perform surgical procedures, as graduates would undergo a 2-year internship to consolidate on-the-job learning in a supervised environment (Figure 2).

On the other hand, students expressed concerns about a lack of clinical exposure, an inability to perform surgical procedures, and limited exposure to surgical emergencies and patients requiring acute surgical care. They felt adequately prepared in theory but had concerns about their clinical competency and skills acquisition, particularly as they entered their internship:

‘No, I was not satisfied because it’s Surgery, and definitely as a final year student, I will not lie, you are hoping that you will get as much clinical exposure as possible. You are hoping that you will get to touch and see. Myself particularly, I learn most when I see something, and I actually do it, and I don’t forget it, as opposed to just hearing it out … Next year I’m going to be an intern, I am quite worried because I have not seen much of surgery, how am I going to navigate that? I worry that I don’t have all the information necessary for me. I know we have the intern booklet, but I am worried about my practical skills.’ (Student A, female, Year 6)

They also expressed concerns about unfamiliarity with surgical instruments and equipment and about not being able to ‘speak the language of Surgery’. They suggested the ability to practice certain surgical skills, such as inserting intercostal chest drains, in a simulated skills laboratory setting, and to be provided with videos of procedures performed in the local context. Student cohorts who rotated through the block later in the academic year seemed more optimistic about their internship because of their exposure to patients on other rotations, such as Family Medicine, where they were placed on the clinical platform and had the opportunity to practice skills like suturing. They were willing to learn and understood that the internship would be a supervised, on-the-job learning experience:

‘So I met up with a couple of previous students who did surgery during pre-COVID conditions, and what most of them would tell me is that even though we don’t have the benefit of practical clinical exposure, we have the benefit of access to lots of theory, and they kept on reiterating that look you guys will get your practice during internship. You will definitely get your practice, and you must take advantage of the theory that you guys are getting, and it’s properly self-directed, you can easily navigate your way through the block.’ (Student B, male, Year 6)

### Module structure and self-organisation

Tutorial schedules for the live Zoom sessions were emailed to tutors a week before the rotation started to allow adequate planning time. The online format afforded them flexibility and the ability to reschedule missed tutorials as a result of emergency clinical commitments (Figure 2). Some tutors found the Moodle platform for asynchronous activities useful because of the ease of setting up quizzes and its accessible, updatable repository of learning content:

‘So, I found Moodle quite useful. Obviously, we don’t know how to use it to its full ability, but the little that we did learn last year, because we were forced to adapt to the online programme quite quickly. It seemed to be quite useful. It’s quite a nice platform. And I am sure if we maybe invest a little bit more time in trying to learn how to use it to its full capacity, we will find it quite useful for teaching – and for examining as well.’ (P4, male, tutor)

Most students experienced the online module in General Surgery, which was well organised and well supported by administrators. They also found the Moodle platform easy to navigate, with content topics appropriately organised by week and videos categorised into clinical skills, procedural videos, past tutorial recordings, quiz sections and past examination papers:

‘I’ve liked the organisation of the programme, because they will make sure that they give you the learning objectives. They will also remind us that at this time, we have to submit this assignment, and the deadline is at this specific period of time. All the lecture materials, yeah, they are there. We were able to access the videos just as my colleagues have said, so we’re able to access the materials, the videos, the slides, and some of the textbooks.’ (Student F, male, Year 6)

However, some students reported being overwhelmed with the volume of resources on the Moodle platform. Some students reported difficulty staying motivated through the block and found the routine monotonous. Others commented that they had developed stronger time-management skills and thought they were becoming better self-directed learners:

‘The challenge that just came to mind was with regard to the resources. Initially, I felt overwhelmed with the amount of resources that we had, and there was a lot of different information from seminars and previous lectures from the current lecture on one topic. There were also videos, and I just felt overwhelmed by the amount of work, the amount of information that was available … but I think the one thing that being in the situation has really highlighted and kind of occurred to me is the importance of asking, is the importance of being able to be honest about what you don’t know, but I think it also taught me that you actually have to do something in order to know what you don’t know and what you do you know, it’s not just enough, you know, and I had to become a more independent learner and self-directed.’ (Student E, female, Year 6)

### Information technology-related challenges

Consultants at central teaching sites were provided with laptops and mobile data to facilitate online teaching. However, junior faculty, including medical officers, who were not in formal teaching posts at the university, had to use their devices and mobile data for teaching, as some teaching sites lacked Wi-Fi in 2021. Most tutors reported that engaging with this module was their first experience with online teaching (Figure 2). They recognised the need to adapt quickly and had become familiar with online software, such as Zoom. The Zoom platform was easy to use, allowing two-way interaction with students via camera and microphone.

However, some tutors found that students would share a single device and access Zoom on their mobile devices, and the small screen limited the quality of their image viewing, such as X-rays. While initial training was provided to faculty by the university’s online learning department, the onus was on faculty to reach out to the IT department for assistance with specific topics, such as Moodle. Another challenge identified was poor internet connectivity, as reported by students, or problems with their microphones; this affected interaction with students, and one of the consultants suggested that students turn on their cameras during sessions, as simply logging on to Zoom was not sufficient evidence that they were present.

Students accessed the online content using laptops and mobile devices. The student residences had Wi-Fi, and students were also provided with mobile data from the university. In most cases, the latter was adequate, except if students had to download large video files. Another issue that affected connectivity and internet speed was the power supply interruption to relieve demand on the electricity grid, a practice known as load shedding in South Africa. Consequently, tutorials would need to be rescheduled; however, having recordings of previous tutorials helped circumvent this issue:

‘Especially during loadshedding, we have a bit of connectivity problem that’s why it’s very useful that the lecture should be recorded so that even if you didn’t have data or connectivity, but you will be able to catch up.’ (Student B, male, Year 6)

Most students used Zoom for the first time, and some of them used it to discuss cases or challenging topics with their peers. One student suggested keeping their camera on during the live Zoom session to encourage participation, while another suggested using polls and breakout rooms for small-group discussions. Some revision sessions or tutorial recordings lasted over 2 h, and students found it challenging to concentrate for that length of time:

‘I think my suggestion would be regarding revisions, for example, the revision shouldn’t go more than five hours, or they should even reach five hours, at least up to three hours or two hours, so that we also from our side I can get time to do our own study.’ (Student B, female, Year 6)

### The way forward post-COVID-19

Tutors were willing to continue with some form of online teaching even after COVID-19 restrictions were lifted. Regarding assessments, there was a suggestion to continue with the case-based scenario testing format, as it was objective, standardised, tested students’ problem-solving skills and aligned with trends in postgraduate examination methods (Figure 2).

Most students recommended that online teaching should continue in the post-pandemic setting. While theoretical content was sufficiently addressed through live Zoom sessions, this should be complemented by students resuming clinical exposure:

‘I think the pre-COVID way of teaching is going out of the window, but I highly support the hybrid model. The fact that you attend hospital from 8-2, then have a Zoom lecture or recording you can watch in your own time, is where we are heading in the future.’ (Student D, female, Year 6)

## Discussion

This study examined faculty and student perspectives on the online teaching and learning module implemented during the 2020–2021 COVID-19 pandemic. The module’s design aligned with adult learning theory and the CoI framework, emphasising social, cognitive and teaching presence (see Table 1).^2,5,8^ Faculty and students reported several positive aspects, including the module’s organisation, accessibility and availability of resources, standardised content and alignment of learning outcomes with teaching and assessment methods. However, challenges were noted, particularly the lack of clinical exposure and procedural skills training, as well as difficulties in engaging students during live sessions. These views are reflected in the five major themes reported in the findings, based on data gathered from FGDs held with faculty and students 1 year after the implementation of the online teaching and learning module.

Student engagement is crucial for academic success, achievement and satisfaction.^27^ This is established through interaction, and in online learning, it includes interaction with content, instructors and peers.^28,29^ The online surgical module at UKZN incorporated these three types of interactions. In a 2020 cross-sectional study exploring students’ perceptions of the online surgical module at UKZN, most students reported having opportunities to ask questions during synchronous tutorial sessions, being comfortable participating in online class discussions and feeling part of a learning community.^1^ To encourage class participation in synchronous Zoom sessions, students suggested that discussions could improve if instructors had a list of students to call on during the live tutorials.^1^ This approach was used by faculty in a qualitative study conducted in the UAE, in which class participation was graded to encourage student engagement during synchronous online sessions.^15^ Altered human interaction in online learning was a common theme in qualitative studies, leading to feelings of isolation.^15,16,30,31^ Feelings of isolation were not identified as a theme in our data, likely because of daily interactive synchronous Zoom sessions, opportunities for small-group work and informal student groups formed to discuss topics.

The flipped classroom pedagogical approach was used to supplement discussions of clinical scenarios. The flipped classroom approach involves assigning teaching materials to be learned outside of class (asynchronously) and utilising live, synchronous online sessions to facilitate ‘engaged and active learning’ through group discussions, reflection and formative assessments.^32^ Case-based and clinical scenario teaching gauged students’ clinical reasoning skills in developing a differential diagnosis, deciding on relevant patient investigations and management, and, with feedback from tutors, reinforced constructivist learning theories, i.e. ‘learning by interaction with the environment’, which, during the COVID-19 pandemic period, was the ‘clinical setting’ reimagined through these modalities.^4^ This approach encourages self-directed learning, a form of experiential learning that makes the learning purposeful and meaningful.

In addition, students’ comments about the importance of grasping concepts reflected a deep-learning approach. This was partly prompted by the teaching methods and the tutors’ dedication on the platform to impart knowledge to students and check their understanding during revision sessions. Students with a deep approach to learning are more likely to be lifelong learners.^33^ Overall, students reported positive learning experiences and satisfaction with the online teaching and learning module. Our findings align with those of similar qualitative studies.^14,16,34^ Tutors’ concerns about limited feedback on student assessment performance highlight the importance of maintaining structured communication between examiners and teaching staff, even as marking has moved online, to enable tutors to adapt their teaching to identified gaps in student performance.

Most students expressed concerns and anxieties about their lack of practical and clinical exposure. Students in the latter part of the year felt more confident as they approached internship, which may reflect their cumulative learning throughout the year, practical exposure in other clinical rotations and greater maturity compared to students from earlier parts of the year. Faculty were cognisant that most students had up to 2 years of pure online teaching in General Surgery, and they were willing to provide further clinical supervision and training once students had graduated. The lack of practical exposure and subsequent preparedness for practice post-graduation were major negative aspects of online instruction in medical and health science education, as reported in similar qualitative studies.^14,16,34^ In a previous quantitative study, 59% (54/91) of students agreed that they had been adequately prepared to apply their knowledge and skills in a clinical setting, but, on analysis of open-ended responses, expressed concerns about the lack of clinical exposure to reinforce their application of the theoretical knowledge in an appropriate clinical setting.^1^ Another gap with pure online methods concerns the formation of students’ professional identities, which depends on face-to-face interaction with clinical teachers and role modelling in these settings.^35^ Students’ suggestions for simulated skills laboratory sessions and locally filmed procedural videos offer a practical, low-cost means of supplementing theoretical online content with procedural exposure and merit consideration in future iterations of the module.

Students reflected on being self-disciplined and demonstrated a sense of agency and empowerment in their learning. They also acquired time management, personal organisation and IT skills. These findings align with other qualitative studies, in which students reported being able to take control of their learning, save resources and review recorded classes as many times as needed.^30,31^ On the other hand, some students reported low motivation, likely because of the monotony of online learning and predictable daily routines. In a German study, fifth-year medical students similarly reported negative effects on their mental well-being and motivation because of the monotonous nature of online instruction.^34^

Technical challenges identified in qualitative studies in medical and health science education included slow internet speeds,^30^ unstable internet connections,^10,15,31^ power cuts^10^ and a lack of access to smart devices.^36^ All students at our university were provided with laptops and mobile data to facilitate online learning. Most reported minimal IT-related challenges. Load shedding was unavoidable and posed the main challenge to accessing live Zoom sessions, resulting in the cancellation and rescheduling of classes. However, access to previous recordings on Moodle mitigated this issue. The reported difficulty in viewing diagnostic images, such as X-rays, on small mobile screens is a device-related limitation particularly relevant to image-based clinical teaching. It highlights a practical consideration for selecting platforms and devices in future online modules.

Another issue identified in qualitative studies was a lack of nonverbal communication between tutors and students, particularly when cameras were off during sessions.^14^ Nonverbal cues, such as eye contact, gestures and posture, foster interaction and connection during synchronous sessions.^14,34^ While faculty and students suggested cameras on in live Zoom sessions, bandwidth constraints and data costs made this impractical for large classes. Some studies have reported that prolonged screen time and prolonged periods of sitting can affect both vision and posture.^10,15^ In our study, no participants spontaneously reported physical discomfort related to online learning. However, some students commented that the length of uploaded videos on Moodle and the revision sessions affected their concentration. They suggested editing the recordings and replacing them with shorter sections on the same topics. Regarding long Zoom sessions, one suggestion to combat screen-time fatigue is to schedule regular breaks.^31^

Both students and faculty identified aspects of the online teaching and learning platform that they valued and were willing to continue with some form of online instruction in the future. Students in our study belonged to the so-called ‘Generation Z’; they demonstrated adaptability to online instructional methods, likely because of their familiarity with technology, including smartphones and social networking sites, for sharing information.^37^ They may have also engaged in other forms of online learning in the past,^1^ and they can be described as advanced users of digital technology.^37^ Faculty members adopting online methods may be because of their familiarity with Zoom, because all postgraduate teaching and academic meetings transitioned to Zoom during the pandemic. This, together with departmental support for online teaching in General Surgery and guidance from the university on policy regarding teaching and assessment processes during this period, facilitated faculty acceptance of online teaching. Although this period was stressful for healthcare professionals generally, lockdown-related reductions in trauma and elective surgical caseloads may have afforded faculty more time to experiment with online teaching tools. In a mixed-methods study, faculty members suggested that 30% of the curriculum be delivered online post-COVID-19, as it was time- and effort-saving; similarly, students found online teaching helpful and complementary to their learning.^23^ In a qualitative study conducted in Saudi Arabia, students expressed a preference for continuing online learning post-pandemic, particularly in fields such as radiology, forensic medicine, scientific research and other theoretical courses.^14^

The main strength of this study lies in its potential to provide an enriched understanding of faculty and student experiences through a qualitative approach. The themes generated are broad, and the findings may be transferable to similar higher education settings in Africa and other middle-income countries. There are some limitations to this study: convenience and snowball sampling methods, that is, approaching the student representative for each rotation to help recruit more students via their social networks, may have introduced selection bias; however, qualitative studies do not aim to be generalisable and thus do not necessitate random or representative sampling.^15^ The lead researcher, Sumayyah Ebrahim, was the module coordinator responsible for designing aspects of the online teaching and learning module, and both Sumayyah Ebrahim and Ruvashni Naidoo were involved in online teaching, which may have introduced bias in favour of positive responses by interviewees or may have sought confirmation of their attitudes and beliefs. However, both conforming and variant views were considered and reported. Because Sumayyah Ebrahim attended all FGDs and was known to participants, this may have introduced response or social desirability bias. We sought to prevent this by conducting FGDs with students after they had completed their rotations. The virtual FGDs were also the best option given social distancing restrictions at the time. However, because most interviews were conducted with the camera off, we cannot comment on participants’ facial expressions, gestures or other nonverbal cues.

These findings have informed ongoing refinements to the General Surgery online teaching and learning module at our university.^38^ Over the past 5 years (2022–2026), the department has adopted a blended instructional approach, incorporating daily large-group Zoom teaching to maintain consistency in delivery.^38^ Students have since resumed clinical placements, engaging directly with patients, participating in surgical intakes and observing or performing selected procedures under supervision.^38^ All video resources, including weekly Moodle LMS quizzes, remain accessible to students, allowing materials to be reused and readily updated.^38^ This model has preserved the benefits of e-learning, including convenience, flexibility, access to resources and opportunities for self-paced, independent study, while adding the value of clinical exposure and experiential learning in the clinical setting.

### Recommendations

Based on our findings, we propose the following recommendations for educational practice and future research:

- Formally evaluate the existing blended instructional model in the General Surgery module, including its long-term impact on knowledge retention, clinical competency and graduate preparedness.
- Sustain and formalise faculty development in online facilitation and digital pedagogy, alongside structured orientation for each incoming student cohort.
- Continue institutional investment in maintaining and updating the existing digital infrastructure (video and resource repository, LMS) to preserve continuity of blended teaching and readiness for future disruption.

## Conclusion

Online learning demonstrated clear educational value, with both groups recognising its advantages in terms of convenience, flexibility and access to resources. Consensus emerged, however, that blended instruction represents the most effective long-term approach, combining online delivery with the irreplaceable benefits of in-person clinical training. The digital library and video repository established through this initiative are now durable institutional assets that enhance routine teaching while remaining deployable during future periods of disruption, including natural hazards, political unrest and conflict. Over time, these resources have become more than a temporary solution to pandemic-related disruption. They now form part of the department’s routine teaching approach and continue to support students’ learning.

As medical education embraces digital transformation, many lessons from this experience may apply to other clinical disciplines exploring blended teaching approaches. Longitudinal studies remain essential for evaluating the long-term impact of blended learning on knowledge retention, clinical skills and graduate competencies, ensuring that curriculum innovation remains evidence-driven and responsive to the evolving needs of students and the health systems they will serve.
